# Peptide Hydrogels and Nanostructures Controlling Biological Machinery

**DOI:** 10.1021/acs.langmuir.3c01269

**Published:** 2023-08-17

**Authors:** Jovana Mitrovic, Gabriella Richey, Sarah Kim, Mustafa O. Guler

**Affiliations:** The Pritzker School of Molecular Engineering, The University of Chicago, Chicago, Illinois 60637 United States

## Abstract

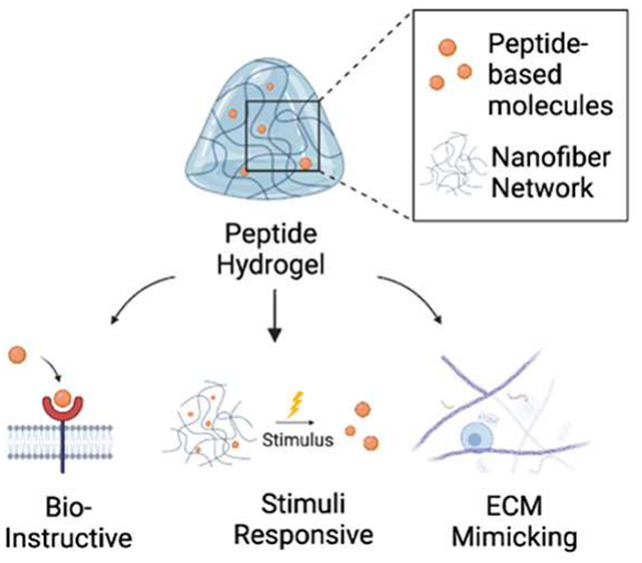

Peptides are versatile building blocks for the fabrication of various nanostructures that result in the formation of hydrogels and nanoparticles. Precise chemical functionalization promotes discrete structure formation, causing controlled bioactivity and physical properties for functional materials development. The conjugation of small molecules on amino acid side chains determines their intermolecular interactions in addition to their intrinsic peptide characteristics. Molecular information affects the peptide structure, formation, and activity. In this Perspective, peptide building blocks, nanostructure formation mechanisms, and the properties of these peptide materials are discussed with the results of recent publications. Bioinstructive and stimuli-responsive peptide materials have immense impacts on the nanomedicine field including drug delivery, cellular engineering, regenerative medicine, and biomedicine.

## Introduction

Synthetic materials are engineered to react to the surrounding biological environment. The bioresponsive materials allow for greater precision and emulation of the biological activity and interactions of natural biological systems. Designing synthetic materials with bioresponsive properties including nanoparticles and hydrogels leads to the development of new tools and methods for a range of biomedical applications, such as drug delivery, immunoengineering, tissue engineering, diagnostics, and imaging.^1,2^ By controlling the features of the synthetic materials, more effective and efficient biomedical technologies can be created with the potential to improve human health and well-being in countless ways.

Protein–protein interactions are fundamental processes in living organisms that have inspired the design of bioactive, stimuli-responsive, and adaptive new nanomaterials.^2^ These interactions play important roles in cellular processes such as signal transduction, cell adhesion, and enzymatic reactions.^3^ By mimicking these interactions, researchers have developed materials that can respond to specific environmental stimuli such as changes in temperature, pH, and the presence of a marker molecule.^2^ The ability to design nanomaterials that respond and adapt to changing stimuli is crucial for the development of advanced biomaterials with diverse functionalities. Peptides provide bioactive cues inspired by protein active sites. They are used in bioactive nanomaterial development, drug delivery, and hydrogels for tissue engineering applications due to their ability to provide bioactive epitopes.^3^ By exploiting and mimicking protein active sites, peptides offer a promising avenue toward developing a wide range of nanomaterials and hydrogel designs for therapeutics and diagnostics. Peptides enable nanoparticle and hydrogel production through the self-assembly process, inspired by β-sheet and α-helical structures in the peptide networks and globular protein structures.^2,4^ By the manipulation of these highly structured peptide complexes, a variety of bioactive nanomaterials can be produced. Peptides can produce nanoparticles with varying sizes and shapes; they can also be used to build three-dimensional hydrogel networks.^4^

Hydrogels, composed of self-assembled peptide nanostructures, are an emerging class of biomaterials, which have unique physical and chemical properties, making them an attractive option for tissue engineering and regenerative medicine.^5^ Self-assembled peptides can form hydrogels that closely resemble the three-dimensional (3D) structure of the extracellular matrix (ECM), providing an ideal scaffold for supporting cells.^4^ The hydrogel scaffold can present physical and chemical cues, including adhesion sites, growth factors, and signaling molecules, to support cellular activities such as proliferation, migration, and differentiation.^5^ Tunable properties of self-assembled peptide hydrogels allow for precise control over the mechanical and biological properties of the scaffold. The use of self-assembled peptide hydrogels in tissue engineering, drug delivery, and biomedical research has shown promising results, suggesting that these materials have significant potential for clinical translation.^6^ Peptide hydrogels can form three-dimensional (3D) tissue models such as organoids, which recapitulate the in vivo architecture and function of organs, providing a powerful tool for studying cellular biology, tissue engineering, disease mechanisms, and drug discovery.^7^ The use of peptide nanostructures for organoid culture has the potential to revolutionize the field of disease modeling and drug discovery, providing a more accurate representation of human biology and enabling the development of more effective therapies.^8^

Peptide nanostructures are engineered to specifically target certain cells and tissues by incorporating targeting ligands on their surface.^9^ Through the conjugation and encapsulation of drugs into these nanostructures, they can be delivered directly to the desired cells and tissues, increasing their efficacy and mitigating adverse side effects.^9^ Peptide nanostructures can also protect drugs from premature degradation and improve their solubility and bioavailability.^10−14^ In addition, immunomodulatory peptide nanoparticles are used for developing vaccines that can effectively trigger the immune system against various diseases.^15^ These nanoparticles are surface-modified with these immunomodulatory signals and are designed to mimic the structure and function of natural pathogenic triggers, such as viruses and bacteria, in order to produce an immune response.^16^ Furthermore, these nanoparticles are tailored to target specific pathogens, making them an attractive option for developing vaccines against a variety of diseases.^17^ In addition, immunomodulatory peptide nanoparticles and hydrogels are also being explored for the development of immunotherapy drugs, which can stimulate the immune system to attack cancer cells.^9^

In this Perspective, we review peptide-based materials forming nanostructures and hydrogels for controlling biological machinery including the extracellular matrix microenvironment, targeted delivery platforms, protein binding, and immunostimulatory systems and present a forward-looking view of the field.

### Extracellular Matrix Components and Hydrogels

**Figure 1:**
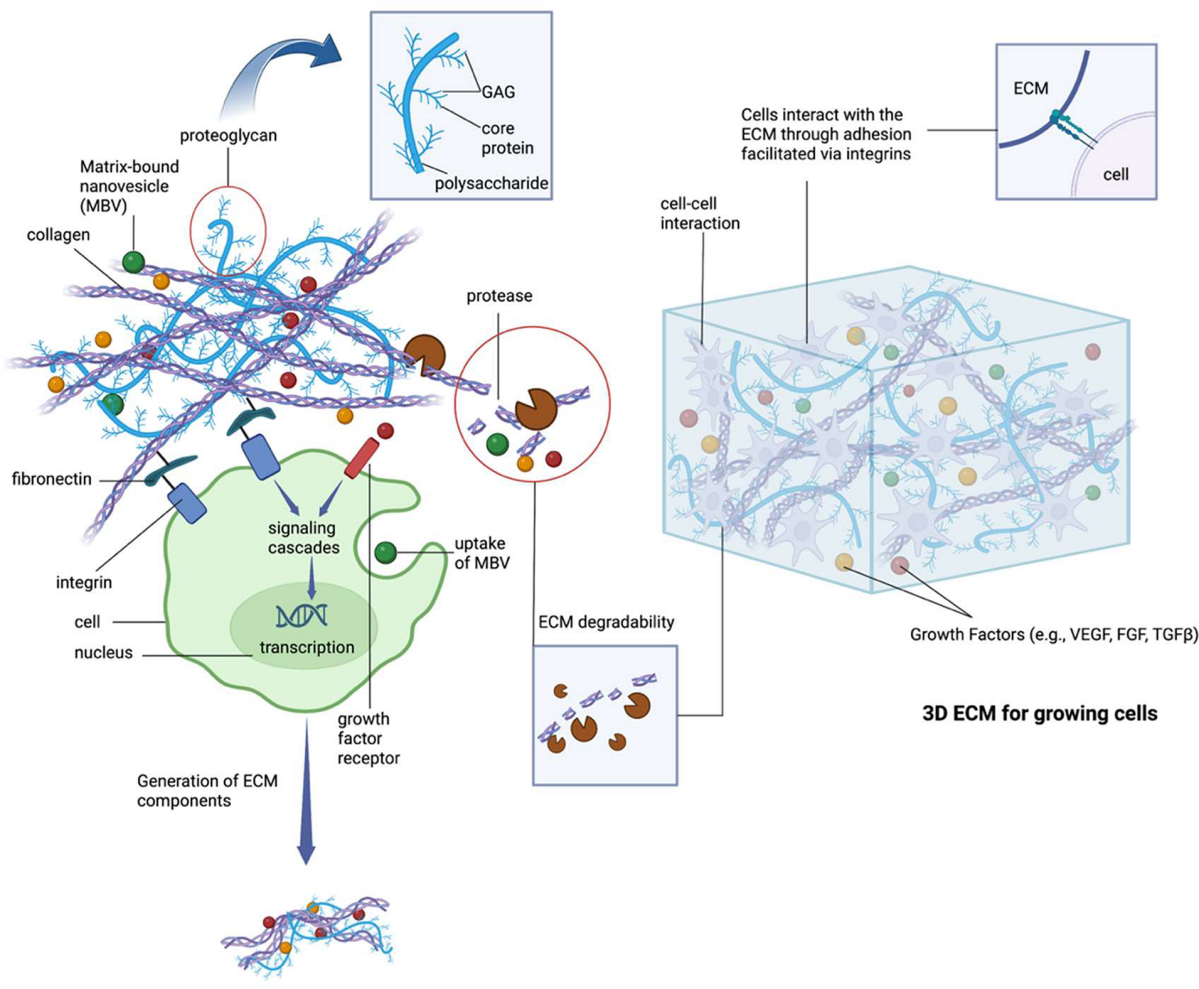
ECM consists of an extensive network of proteins and molecules surrounding and affecting cells. ECM plays an essential role in cell–cell interactions, interacts with cells through adhesion facilitated via integrins, and influences cell growth, movement, differentiation, morphogenesis, and homeostasis. ECM is degradable via protease, and new ECM components are generated via a signaling cascade through growth factors and integrin receptors. Matrixes that mimic ECM are exploited as cell culture scaffolds for growing cells. Created with BioRender.com.

The extracellular matrix (ECM) is a noncellular three-dimensional network surrounding all tissues that provides structural and functional support. ECM plays a vital role in many cellular processes, such as homeostasis, differentiation, morphogenesis, cell growth, and movement, in addition to structural or physical function.^18,19^ The overview of the regulation and features of the ECM in cellular processes is shown in Figure 1. Proteoglycans (PGs) and fibrous proteins such as collagens, elastins, laminins, and fibronectins make various tissue-specific ECMs.^19^ Proteoglycans have hydrating and buffering properties that allow them to form a force-resistant material that looks like a hydrated gel.^19^ Glycosaminoglycan (GAG) chains are covalently linked to a core protein to form proteoglycans.^20^ Collagen is the most abundant protein in the body and the most abundant fibrous protein in ECM. Collagen supports ECM through providing tensile strength, facilitating cell-to-cell adhesion and migration.^18^ Another structural protein, elastin, provides elasticity and stretch through its unique recoil characteristic. Fibronectin binds to various components of ECM such as integrins and plays an important role in adhesion, migration, growth, and differentiation.^18^ Laminins are key components of the basal lamina with important functions in structural support, cell adhesion, and filtration in the glomerulus of kidneys. The ECM is a crucial structure in the human body necessary for its function. The cause of many disorders and fatal diseases is the degeneration of the extracellular matrix. Developing biomaterials that can mimic ECM as effectively as possible widens the possibilities for applications for biomedical and tissue-engineering purposes.

The ECM-mimicking hydrogels provide three-dimensional structures formed via the cross-linking of hydrophilic network materials. Natural or synthetic polymers can be physically or chemically cross-linked to form biomaterials, and their structural composition enables them to mimic the ECM and provide a synthetic platform for biomedical and tissue engineering applications. Peptide hydrogels are composed of 3D fibrous networks that closely mimic the ECM and can be used for biomedical, tissue-engineering, drug delivery, and regenerative medicine applications due to their low toxicity and biocompatibility.^21^ Peptides self-assemble to form hydrogels due to various chemical and physical interactions, causing a fibrous network with a highly microporous structure and high water content.^1^ In recent years, there has been an emphasis on using peptide hydrogels due to their tunable properties that can be suited to fill the specific needs for designing complexes for various biomedical applications and drug delivery systems. In addition, peptide hydrogels can respond to external stimuli such as temperature, pH, ions, light, and mechanical stimuli, making them ideal candidates for mimicking native ECM. Recently, Coulter et al. presented an in situ-forming peptide hydrogel as an injectable platform for the delivery of antiretroviral drugs for treating HIV/AIDS.^22^ Peptide hydrogels can be used for drug delivery with an efficient and precise therapeutic effect. The peptide and drugs can be covalently or noncovalently conjugated to the hydrogel system, absorbed by the matrix, or physically entrapped.^10^ It is vital to understand the forces of interaction between the drug and hydrogel network, to understand the release kinetics, and therefore to adjust and manipulate the release kinetics. The size of the drug and the density of the peptide nanofibers are two of the main factors influencing release from a hydrogel. Larger proteins tend to remain in the hydrogel for a longer time compared to smaller proteins.^10^ In addition, the release rate can be increased by decreasing the fiber density and increasing the pore size in the hydrogel and vice versa.^10^ The porosity of the hydrogel influences the diffusion of the proteins. Similarly sized proteins tend to remain longer in a hydrogel with smaller pores than in a hydrogel with larger pores. Tunable properties of hydrogels, such as porosity, allow for the controlled release of molecules, making them superior to other less-tunable carrier systems.

Peptide hydrogels can be modulated to mimic the biochemical and mechanical properties of the ECM, leading to more effective cellular treatments. Short peptide sequences have been identified and derived from the ECM to support cell adhesion and growth. These sequences can be incorporated into the material design to promote cell–cell and cell–matrix interactions, which directly influence cell behavior.^23^ These materials utilize peptide functionalization to mimic the cellular environment and provide molecular cues to promote tissue regeneration and repair.^24^ These short peptides are more stable and exhibit less steric hindrance compared to their full-length proteins.^25^ The fibronectin-derived cell adhesion sequence arginine-glycine-aspartic acid (RGD) has been demonstrated to promote cell proliferation in vivo and in vitro.^23,26^ Laminin-derived peptides can be functionalized to induce cell aggregation and adhesion.^27^ Laminin-derived peptides include IKVAV, YIGSR, PDSGR, and RYVVLPR.^28^ The use of SIKVAV (Ser-Ile-Lys-Val-Ala-Val) in PHEMA hydrogels has been shown to increase the proliferation and adhesion of mesenchymal stem cells in vitro.^29^ We previously demonstrated the use of a laminin mimetic peptide (LM/E-PA) to emulate the structure and function of laminin to promote satellite cell activation to induce the repair of skeletal muscle tissue.^30^ Collagen-derived peptides can be utilized to imitate collagen’s structural and signaling functions by forming collagen-like bundles or motifs that resemble collagen, including peptides such as DGEA, FPGER, and GFOGER.^28^ The presentation of DGEA in MSC hydrogels has been previously shown to induce the osteogenic phenotype and has been investigated in bone tissue engineering.^31,32^ Glycosaminoglycans (GAGs) are another important structural component of the ECM. GAGs retain large amounts of water; this retention renders the ECM resistant to mechanical forces and is especially important in load-bearing tissues such as cartilage.^33^ We previously investigated heparin mimetic peptides (HM-PA, lauryl-VVAGEGD(K-psb)S-Am) that mimic heparan sulfates. HM-PA nanofibers demonstrate better binding profiles to VEGF, hepatocyte growth factor, and fibroblast growth factor-2.^34^ The utilization of HM-PA nanofibers in a hydrogel system demonstrated increased angiogenesis, wound closure, and re-epithelization in burn wounds.^35^ The use of ECM-mimicking peptides in synthetic materials holds promise as a means of controlling cellular processes for more effective tissue engineering applications.

### Chemical Functionalization and Bioconjugation of Epitopes

The driving force of peptide hydrogels is the peptide sequences themselves, where the functionalization of these peptides directly influences the hydrogel’s properties. Functionalization of these self-assembling peptides involves the addition of various chemical groups such as lipids, drugs, fluorescent groups, and protein binding units. Functional units can be covalently conjugated, where a chemical functionality is used to attach the desired molecule to the reactive amino acid side chains. Amino acids with a chemically functional side chain are used for specific chemical reactions that can be harnessed for nanostructure fabrication. Just as amino acids are the building blocks for peptide chains, so are peptides the building blocks for biomaterials with wide-ranging physicochemical functionalities.^28^ Both natural and synthetic amino acids are used to construct these peptides, and due to the diversity of available amino acids, peptide structures are granted an unusually high degree of tunability. The versatility of self-assembled peptide nanostructures depends on sequence modifications that can change their target cell or organ, impede enzymatic degradation, impart stimulus-responsiveness, or conjugate with other organic or inorganic molecules.^36^ Peptide self-assembly is a spontaneous process driven by a hydrophobic effect, van der Waals forces, hydrogen bonding, and electrostatics. Imaging probes, carbohydrate units, oligonucleotide sequences, and other small molecules can be conjugated to these peptide chemical scaffolds. Incorporation of peptide epitopes in synthetic materials allows for improved cellular communications.^28^ By the incorporation of these changes, biomaterials can control their bioactive processes in tissues. Certain epitopes could trigger the immunological response in a host system.^28^

### Stimuli-Responsive Hydrogels

**Figure 2:**
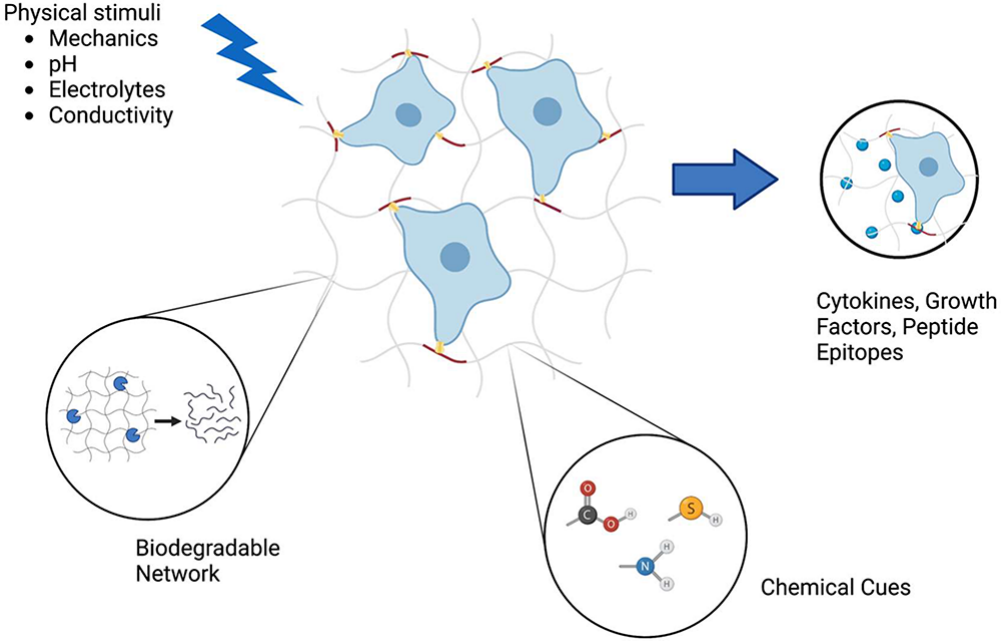
Stimuli responses such as pH, temperature, light, mechanical properties, and chemical functionalities can be tailored for effective biomaterial design. Manipulation of these stimuli can lead to changes in the size, shape, and functionality of peptide hydrogels. Created with BioRender.com.

Hydrogels are polymeric networks made of large hydrophilic peptides that can maintain their structural integrity while becoming saturated with water. They have high water content and tissue-like elasticity and can easily transport nutrients and waste in and out of their matrix, making them ideal mimics of the cell’s ECM.^21^ Their optical clarity allows for nondestructive imaging of the cell function within. To fabricate a hydrogel, liquid precursors must be transitioned into solid-like material through either noncovalent (physical) or covalent (chemical) cross-linking. Most peptide-based hydrogel systems are formed through self-assembly by physical cross-linking.^37^ These peptide hydrogels assemble and form gels with different shapes via hydrophobic charges and electrostatic interactions.^38^ Chemically cross-linked hydrogels may be formed by using redox reactions or photoinitiation to induce the covalent reaction of different chemical side groups for rapid formation.^39^ Collagen contributes tensile strength to the ECM in physiological environments. Collagen-based hydrogels, as a result, have a limited stiffness range. Collagen hydrogels developed using compression techniques had an estimated break force of 0.216 N and a mean tensile break strength of 0.6 ± 0.11 MPa with a modulus of 1.5 ± 0.36 MPa.^40^ This plastic compression method has been shown to promote cell proliferation and migration. The collagen scaffolds have been used to demonstrate that invasive cancer cells can switch from a mesenchymal to an amoeboid motility pattern.^41^ This process is driven by the inhibition of integrin or of the proteolytic activity of matrix-metalloproteinases.^41^ Environmental stimuli including pH, electrolytes, temperature, and light are exploited for manipulating the size, shape, and function of the peptide nanostructures. Stimuli-responsive hydrogels are a smart nanostructured design that allows for accurate mimicking of a natural, biological tissue environment. Tailored features and characteristics allow for a more advanced approach to various biomedical, regenerative, and tissue engineering approaches.^42^ Temperature, pH levels, ionic strength, chemical stimuli, mechanical stimuli, and light are some of the stimuli that could influence the hydrogel and its effectiveness.^43^ These design considerations are summarized in Figure 2. Previously, pH-responsive OE peptide-containing hydrogels have been investigated to release antitumor drugs.^44^ Drug release in these hydrogels is initiated by the acidic tumor microenvironment, inhibiting tumor growth, and minimizing off-target effects.^44^ In another study, temperature-responsive protein hydrogels were developed by combining four peptide blocks to create a controllable sol–gel transition with tunable mechanical properties. The hydrogels were functionalized with RGD peptides and heparin-binding angiogenic growth factors to promote proangiogenic activity.^45^

### Three-Dimensional (3D) Bioactive Environment

Recent advances in organoid technology enable the development of new and possibly more efficient ways of studying disease mechanisms, drug discovery, regenerative medicine, and other biomedical applications. Animal models have been extensively used to conduct in vivo experiments. In addition to the fact that experiments often have harmful consequences for animals, in many cases, the animal bioactive environment does not accurately represent the characteristics of human tissue and the organ environment. The need for developing models that can mimic structural and functional aspects of human tissues and organs in vivo influenced the development of organoids. Organoids are a 3D multicellular bioactive environment derived from stem cells. Organoid models can be formed either from pluripotent stem cells (PSCs) or adult stem cells (AdSCs).^46^ Stem cells are the precursors of all cells, and due to their ability to differentiate, they represent an ideal candidate for bioprinting. Three-dimensional bioprinting is a technique that uses biological materials and cells to produce structures and tissues for the purposes of regenerative medicine and tissue engineering. Various types of organoids can be established and are growing, from tissue samples to whole organs such as the stomach, intestine, pancreas, kidney, brain, etc.

**Figure 3:**
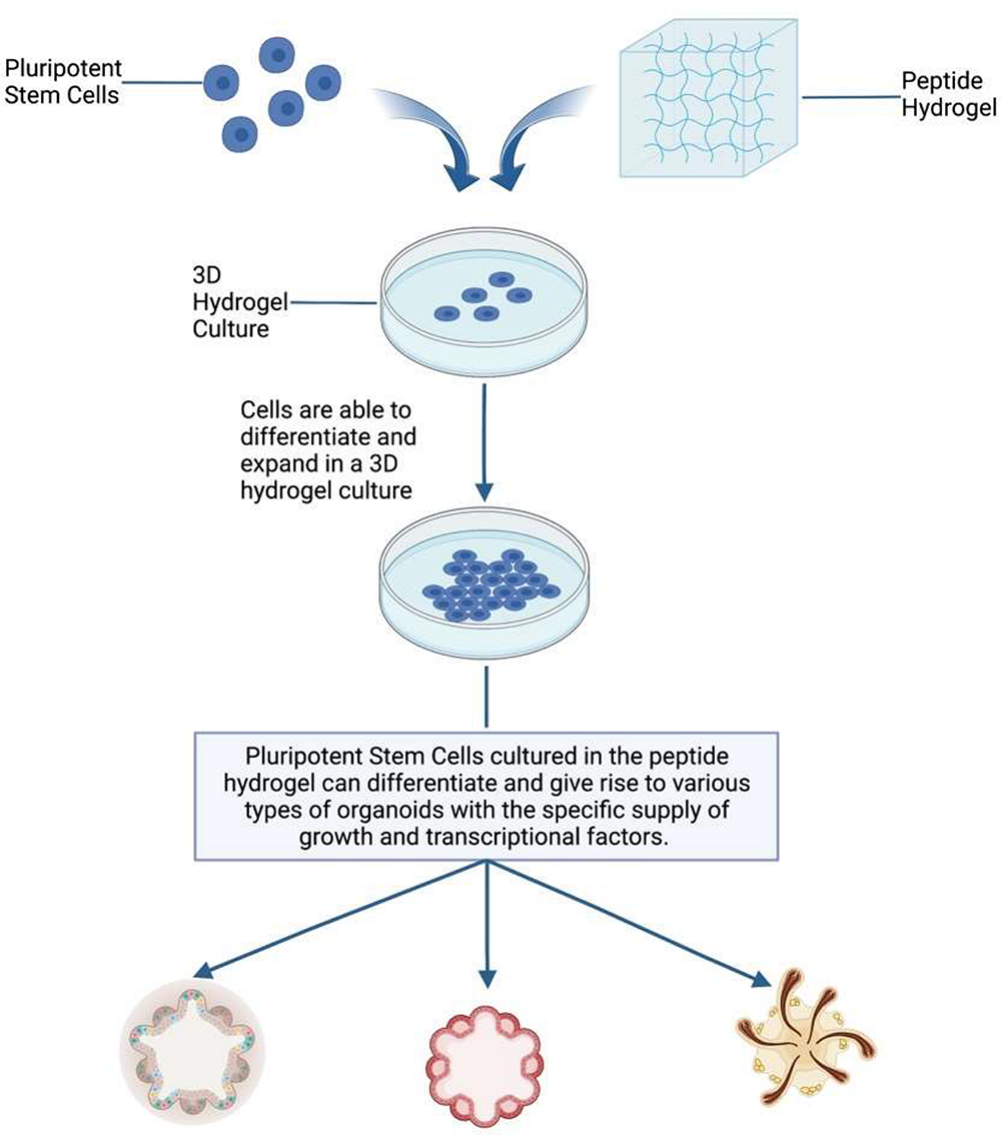
A three-dimensional hydrogel platform is suitable for culturing stem cells. Stem cells can differentiate and give rise to the intended organoid when they are provided with specific growth and transcriptional factors necessary for cell differentiation. Created with BioRender.com.

The natural microenvironment in which stem cells reside or the stem cell niche is an environment that promotes and regulates stem cell function. It is necessary to provide stem cells with an environment that can mimic the in vivo cell niche for isolated stem cells to have the ability to differentiate and self-renew in vitro. When stem cells are embedded in a matrix that mimics the stem cell niche, differentiation, proliferation, migration, and selection occur and are activated by signaling pathways. From cultured cells, organoids can grow into complex structures and mimic the organization of in vivo tissues or organs. For many years, standard practice for developing organoids and growing cells involved Matrigel as a cell culture platform. However, the complexity that includes Matrigel and a poorly defined system has led to an increasing demand for the use of Matrigel-free methods that would enable organoids’ successful and more precise development. Therefore, in recent years, peptide hydrogels have been emphasized as a possible, more efficient method for growing cells due to their unique function of mimicking the extracellular matrix. The overview of the concept of culturing cells in hydrogel matrixes is shown in Figure 3. For stem cell culture to be successful, the matrix in which the cells are located must mimic the stem cell niche and its factors that stimulate cell differentiation. In vitro, it is necessary to deliver and control certain factors that would stimulate the development of stem cells into organoids. However, the complexity of Matrigel makes the control and timely delivery of factors difficult. One of the reasons for this complexity is that Matrigel contains more than 1800 unique proteins as determined by proteomic analysis, making Matrigel more susceptible to variations.^7^ Also, there may be some necessary components for growing organoids missing in the Matrigel, such as laminin-511 and the absence of mesenchymal cells shown when growing intestine organoids in Matrigel, resulting in different architectural design and structure formation than for its in vivo pair.^7^ The use of Matrigel includes limitations such as culture reproducibility and clinical translation due to batch-to-batch variation and the origin of the matrix that comes from the mouse tumor environment.^47^ In addition, due to the source from which Matrigel is made, organoid transplantation into the human body is limited due to immunogenicity. Also, due to its complexity, it is difficult to tailor Matrigel to accommodate the unique environments of different organoids.^47^

Peptide- or protein-based hydrogels have been explored as possible materials for growing organoids that can overcome issues involved with Matrigel and provide better tunable properties. Many alternative materials have been tested for intestine organoids. Collagen I hydrogels were used to culture murine intestinal organoids^47^ and human stomach, intestinal, and colonic organoids.^7^ Also, a human colorectal carcinoma model was developed using collagen I hydrogels in a culture of rabbit colons.^7^ Curvello et al. investigated the way to improve the mechanical properties of collagen I hydrogels for a culture of intestinal organoids by adding nanocellulose, and they were able to develop a thermoresponsive matrix whereby adjusting the collagen/nanocellulose ratio allowed the gel stiffness to be controlled.^48^ More complex hydrogel structures have been explored for developing organoids for studying disease mechanisms. Silk fibroins and collagen, in combination, have been used to develop an intestinal organoid to which bacteria such as *Escherichia coli* can be introduced and the immune response monitored.^47^

In a similar vein, three-dimensional models have been increasingly applied to cancer research.^49^ These in vitro tumor models, aptly named “tumoroids”, are excellent platforms for testing chemotherapeutic agents against cancer metastases.^40^ The construction of 3D osteosarcoma tumoroid models serves as useful prognostic tools, especially for patients who have responded poorly to standard care and are either at risk for disease progression (metastasis) or have already progressed. Osteosarcoma, as well as other rare cancers, has historically been less researched for novel drug discovery and preclinical model development. This 3D osteosarcoma model was the first to create a geometrically compartmentalized model built from collagen hydrogel matrixes.^40^ Interestingly, when both basic tumoroids and complex (bone granule-supplemented) tumoroids were exposed to doxorubicin, the standard of care drug for osteosarcoma, the complex tumoroids demonstrated greater cell death.^40^ As the complex tumoroids are meant to reproduce aspects of in vitro cancer behavior, the clinically effective threshold for chemotherapy can be determined more accurately using these systems.

## Moving Forward

### Structure–Bioactivity Relationship

Controlling the size and shape of materials is instrumental in manipulating the fate of the cells and developing new therapeutic methods. Molecular mimicry of the extracellular matrix in hydrogels is necessary to provide the correct cellular cues to promote bioactivity. As previously summarized, the use of ECM-mimicking peptides in biomaterial scaffolds provides a means to control the cell fate. Effective tissue engineering and repair require exploiting these cell–matrix interactions. More effective material designs for tissue engineering and repair should incorporate these peptides into biomaterial designs to promote bioactivity. Additionally, controlling the stiffness of the material has also been shown to play an important role in stem cell commitment.^50^ Mechanical cues have also been shown to impact cell fate and its rate of proliferation in 3D networks.^51,52^ Controlling these properties in the material can further influence stem cell commitment and proliferation rates in 3D networks. To control the stem fate, peptide amphiphiles are used to self-assemble into nanofibers with tunable mechanical properties, allowing for the creation of peptide hydrogels with a range of size, shape, and mechanical properties. Peptide amphiphiles are a class of molecules composed of four parts: a hydrophobic site, a short peptide sequence that forms a β-sheet, a hydrophilic peptide sequence, and a bioactive peptide group.^53^ This versatile structure allows peptide amphiphiles to self-assemble into nanofibers, which can form the basis of peptide hydrogels with tunable properties. By controlling the sequence and composition of the peptide and hydrophobic segments, the size, shape, and mechanical properties of the resulting peptide hydrogel can be altered.^53^ In addition, the structure of such peptide networks should be strongly considered in functional material designs. Specifically, peptide scaffolds with porous structures and a high surface area to volume ratio provide a suitable environment for cells to adhere. Such environments promote material exchange between cells and their environment.^54^ Hydrogels provide a unique capacity to be modulated to fit the needs of their biological component. Consideration should be taken to incorporate these design parameters into more effective materials for tissue engineering.

### Bioinstructive Materials and Protein Binding

Tissue engineering aims to elicit cell behavior in response to a biomaterial. Such processes rely on incorporating bioinstructive signals to elicit desired cellular behavior. Bioinstructive peptide networks can coordinate various cell processes such as cellular proliferation, differentiation, and tissue regeneration.^3^ The integration and design of these peptides depend on the application and tissue type of the material. For example, QHREDGS (Q) peptide hydrogel has been shown to improve wound healing by increasing keratinocyte migration and the stimulation of neovascularization.^55^ Recently, the collagen-mimetic peptide GFOGER has been used as a chondrogenic inducer.^56^ Incorporation of this peptide into hydrogel resulted in the retention of bone marrow-derived mesenchymal stem cells, providing a platform for further research into osteochondral regeneration.^56^ Using scaffold and support materials is important for diversifying the function and properties of the peptides. In addition, the presentation and intensity of the epitopes presented in peptide networks play a crucial role in determining cell behavior.^3^ The use of multiple epitopes in these structures can coordinate multiple bioactive signals to better regulate the cell activity. For example, RADA16 peptide nanofiber has advantages in providing an ECM-like environment for promoting cell attachment and proliferation. Utilization of this scaffold in combination with a copper peptide glycyl-histidyl-lysine (GHK) functionalized the system to also promote angiogenesis and accelerate diabetic wound closure.^57^ The design and integration of bioactive peptides into scaffold materials should take into consideration the application of the material, tissue type, and presentation of epitopes presented in the peptide network.

### Living Hydrogels for Organoids, Transplantation, In Situ Forms of Tissue, and Regenerative Medicine

**Figure 4:**
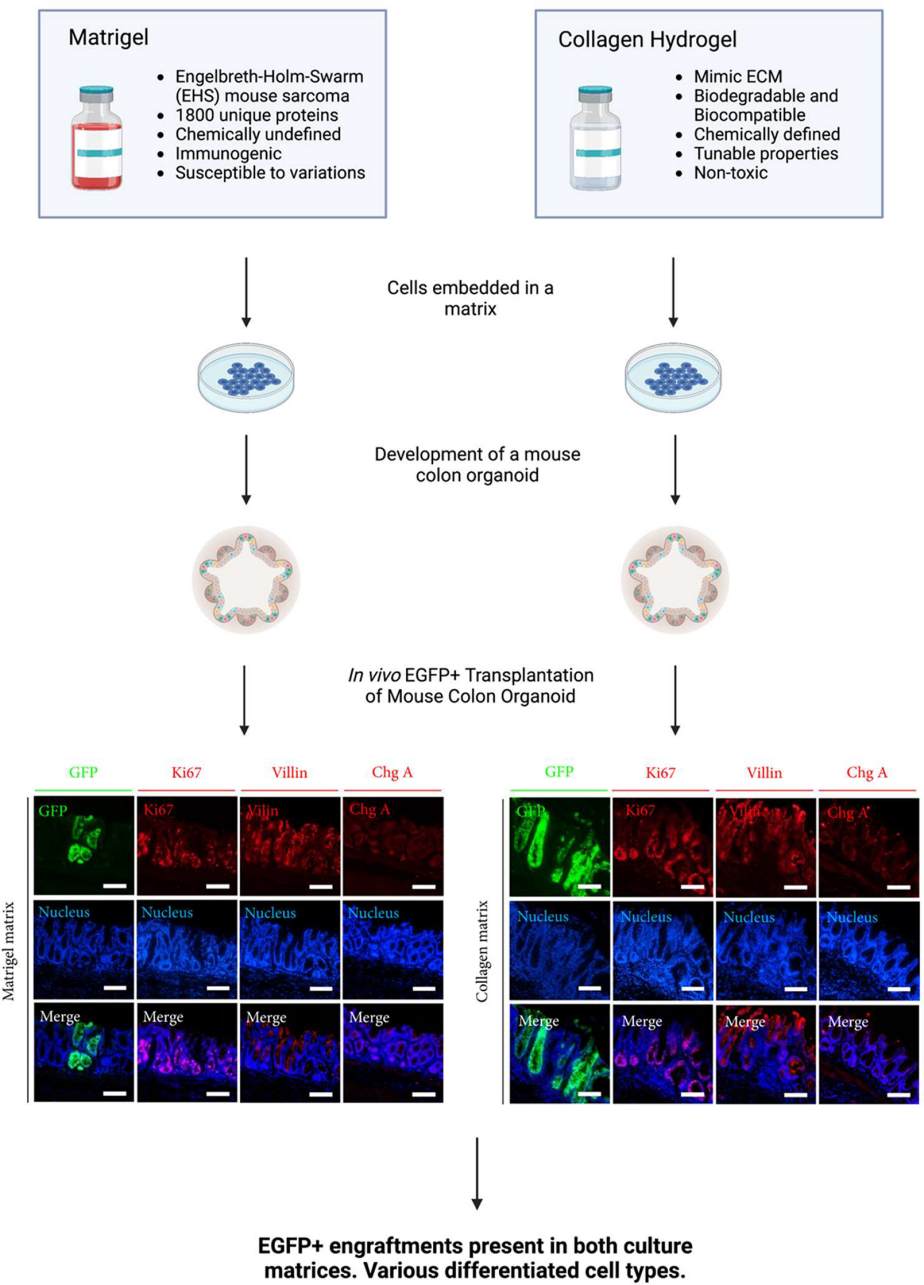
Overview and comparison of Matrigel and collagen hydrogel properties. Both matrixes are suitable for culturing cells for developing organoids. Colonic organoids cultured in both matrixes transplanted into an injured mouse model showed engraftment and cell differentiation via immunocytochemistry analysis. The superiority of the collagen hydrogel nature and safety compared to Matrigel shows potential for growing organoids in vitro suitable for transplantation and regenerative medicine. The figure is partially adapted with permission from reference (59). Copyright 2019, the authors.^59^ Created with BioRender.com.

The production of laboratory-grown living organ constructs remains a major goal of regenerative medicine. Tissue transplantation is extremely limited by a deficiency in donor organs, creating a strong need for engineered organs. Advances in the organoid field have demonstrated the necessity to harness cell-driven organization to mimic organ-like behavior.^8^ Providing an environment for cells to retain their biological functions is essential for creating these systems. Conventionally, animal- or tumor-derived matrixes have been used to develop organoids. The use of such matrixes limits the application of organoids for regenerative purposes. As mentioned above, due to the host’s immune response, there is a considerable risk in using organoids developed from matrixes such as Matrigel for clinical transplantation. To realize the full potential that organoid design offers, it is necessary to perfect the engineering of materials for a 3D cell culture. Due to their unique characteristics of biodegradability, biocompatibility, and highly tunable chemical and physical properties, hydrogels can mimic the ECM in the most suitable way and become an increasingly promising candidate for organoid development, clinical transplantation, and regenerative medicine. Collagen and other naturally derived materials (chitosan, alginate, and hyaluronic acid) and polymer-based hydrogels have experimentally shown promising results.^58^ Jee et al. grew intestinal, stomach, and colonic organoids in collagen hydrogels and compared them to Matrigel-grown organoids, proving the differentiation of cells in both matrixes.^59^ Furthermore, EGFP expressive crypts containing differentiated cell types in the colonic epithelial tissue of the mouse model were found after in vivo transplantation of mouse colon organoid culture from isolated colonic crypts from CAG-EGFP mouse colon to the colon of an EDTA injury mouse model, proving the clinical translation potential of hydrogels as culture matrixes.^59^ Although Matrigel has long been used for cell culture, hydrogels have promising results and are crucial to realizing clinical transplantation applications and using organoids for regenerative purposes. The overview and comparison of Matrigel and collagen hydrogel properties as scaffolds for developing organoids are shown in Figure 4. The precisely defined nature of hydrogels and nontoxicity allow us to imagine a future where the in situ formation of tissues and organs will be possible, as well as transplantation of structures formed in vitro, and where the risk for the patient will be minimized to the greatest extent. In addition, the clinical application of large-scale organ systems relies heavily on efficient vascularization. Functional vascularization remains a bottleneck for the building of complex tissues. One solution to this is through encapsulation of endothelial cell tissue constructs before implantation. This technique previously demonstrated high cell viability and the formation of anastomosis with host vasculature after implantation.^60^ The use of hydrogels with perfusable channels can also help improve the survival of transplanted organs, providing functional vasculature to support the development of tissue in vivo.^61^ The use of hydrogels for organoid development and clinical transplantation is a promising and necessary avenue to meet the demand for laboratory-grown living organ constructs.

### Nanostructures for the Targeted Delivery of Precision Therapeutics

Peptides and nucleic acids are some of the strongest tools for developing and delivering precision therapeutics. However, the macromolecular composition of these tools makes the effective delivery challenging.^62^ Oligonucleotide-based therapies have demonstrated strong promise for disease intervention at the molecular level, but their use is dependent on chemical modification to improve stability. Such modifications improve the stability of the molecule but have limited uptake into cells due to poor membrane permeabilization.^63^ Various nanostructures, including liposomes, dendrimers, and nanoparticles, have been explored for the delivery of nucleic acid-based precision therapeutics. Liposomes, for example, have been used to encapsulate miRNAs for targeted delivery to colorectal cancer cells, leading to significant tumor suppressive effects.^64^ Dendrimers and nanoparticles have also been explored for gene therapy delivery due to their high biocompatibility and ease of surface modification.^65^ Xiong et al. previously used functionalized dendrimer-entrapped gold nanoparticles (Au DENPs) as a nonviral vector for the delivery of plasmid DNA to inhibit cancer cell metastasis in vitro.^66^ Hydrogels make an attractive platform to exert spatiotemporal control of oligonucleotide-based therapies on a macroscopic level. Recently, hydrogel encapsulation of miRNAs functionalized with SKPPGTSS peptide successfully regulated senescence genes while expanding chondrocyte pools to alleviate osteoarthritis.^67^ Physical properties and the type of nucleic acid in these systems can be tuned for other applications including wound healing and myocardial repair.^68,69^ Cell-penetrating peptides (CPPs) have great potential for the delivery of precision therapeutics. The CPPs are positively charged peptide sequences that function to protect their cargo from degradation and facilitate cellular uptake. However, it is essential to design CPPs to enhance membrane permeabilization while reducing nonspecific interactions. We have previously demonstrated the use of arginine-rich cell-penetrating peptides and a proteoglycan binding peptide, KRSR, to increase the membrane penetration of an oligonucleotide-based drug with low toxicity and off-target effects.^70^ Other nanostructures have also been explored for the delivery of precision therapeutics. Further research into the application of hydrogels and CPPs for the delivery of precision therapeutics should aim to characterize the in vivo effects of RNA-based hydrogel systems and understand the host response to them. As the field of precision medicine continues to advance, the development of effective delivery strategies will be critical to their successful translation into clinical use.

### Materials for Immune Modulation

Immunomodulatory therapies offer immense potential for modern medicine in the fields of cancer, autoimmunity, and infectious diseases. However, despite their immense potential, these therapies suffer from systemic and off-target adverse effects.^71^ Therefore, interest has developed in utilizing biomaterials for sustained release and local delivery of immunotherapeutics.^71^ Hydrogels have been previously utilized to release small molecules and stimulate the innate immune system. The slow release of STING agonists from an encapsulating hydrogel matrix triggered innate immune system stimulation and promoted lymphocyte infiltration, resulting in decreased tumor recurrence in cancer models.^72^ Such methods offer a promising outlook for utilizing hydrogels and other biomaterials to increase the safety and efficacy of immunomodulating therapies. Additionally, immunomodulatory peptide nanoparticles have been developed for effective vaccine delivery, overcoming limitations of current vaccines such as excessive reactogenicity.^73^ These materials have modularity, multivalency, and biocompatibility that allow them to function as self-adjuvating vaccine delivery vehicles, improving humoral and cellular immune responses.^73^ The wide range of amino acids allows for tailored design and modification, leading to vaccines and immunotherapies for specific diseases or generalizable platforms for preventing or treating various diseases. Our previous research has focused on investigating the effectiveness of peptide nanofibers containing CpG ODNs in driving the immune response toward a Th1 phenotype, which we found to be more effective than nanospheres.^74^ Previously, Q11 peptide nanofibers were able to act as an effective adjuvant and elicit a robust CD8+ T cell response, protecting mice from a challenge with influenza.^75^ More recently, a multiepitope antitumor vaccine utilizing supramolecular α-helical peptide nanofibers generated strong antitumor effects in mice by engaging both innate and adaptive immune responses.^76^ These findings highlight the potential of biomaterials for increasing the safety and efficacy of immunomodulatory therapies and developing effective vaccines against various diseases.

## Conclusions

We have discussed the bioactive peptide-based biomaterials for tissue engineering, drug delivery, and immunomodulation to manipulate the fate of cells and develop new therapeutic methods. The use of hydrogels in tissue engineering is promising due to their biodegradability, biocompatibility, and tunable chemical and physical properties. Molecular mimicry of the ECM is necessary to provide the correct cellular cues to promote bioactivity. Peptides can be incorporated into new designs to further promote bioactive molecules and control cell behavior. Bioinstructive and stimuli-responsive peptide materials are becoming increasingly prevalent in biological engineering, as they provide a versatile platform for designing complex structures with tailored biochemical and biophysical properties. Furthermore, the ability to precisely control the presentation of these peptides on the material surface allows for precise control over cell behavior and tissue growth. Future research in this area will undoubtedly continue to refine and expand these approaches with more controlled structure and function relationships, potentially leading to more effective and personalized treatments for patients.
